# A systematic review of outcomes following residential treatment for eating disorders

**DOI:** 10.1002/erv.2733

**Published:** 2020-03-20

**Authors:** Tina Peckmezian, Susan J Paxton

**Affiliations:** ^1^ National Eating Disorders Collaboration Sydney Australia; ^2^ School of Psychology and Public Health La Trobe University Melbourne Australia

**Keywords:** eating disorder, outcome, residential, treatment

## Abstract

**Objective:**

Residential centres for the treatment of eating disorders are becoming increasingly common, yet data following residential care are scarce. We reviewed outcomes of residential treatment for eating disorders across all diagnoses, age groups and genders. A secondary goal was to identify treatment elements and patient characteristics that predicted a greater response to treatment.

**Method:**

Peer‐reviewed studies published in the last 20 years were identified through a systematic search of the electronic databases PubMed and Cochrane Library.

**Results:**

Nineteen open‐label studies reporting changes between admission and discharge were included in this review. Most took an eclectic approach to treatment, integrating elements from several different techniques without a unifying theoretical framework. All studies reported improvements in most outcomes at discharge, including changes in eating disorders psychopathology, weight, depression, anxiety and quality of life. Eight studies reported outcomes at some interval after discharge, with largely positive outcomes.

**Conclusions:**

While residential care was associated with consistently positive outcomes, the variability in program characteristics and poor quality of research designs prevent firm conclusions from being drawn about their efficacy. Future research should include controlled studies that evaluate specific theoretical approaches and program elements, include long‐term follow‐up, and compare residential care to other treatment settings.

## 1 INTRODUCTION

Eating disorders are complex mental illnesses associated with a high level of impairment and significant socio‐economic costs (Filion & Haines, 2015). Eating disorders are one of the 12 leading causes of hospitalisation due to mental health issues in Australia (Deloitte Access Economics, 2012), and are associated with high levels of treatment dropout (DeJong, Broadbent, & Schmidt, 2012), relapse (Khalsa, Portnoff, McCurdy‐McKinnon, & Feusner, 2017) and mortality (Fichter & Quadflieg, 2016). Recommended treatment approaches vary depending on disorder and age group, but focus on family‐based therapy with adolescents and cognitive behavioural therapy approaches with adults (Hay et al., 2014; National Institute for Health and Care Excellence, 2017).

Treatment for eating disorders is typically delivered on a continuum of care, starting with outpatient treatment, and moving on to intensive outpatient, day treatment or partial residential, residential and inpatient hospitalisation (Anzai, Lindsey‐Dudley, & Bidwell, 2002). A patient's journey through levels of care is unique, constantly changing and dependent on a myriad of factors such as treatment availability, symptom severity, medical status, motivational status, treatment history and financial constraints (Kaplan, Olmsted, Carter, & Woodside, 2001; Yager et al., 2014).

The distinction between residential and inpatient treatment is largely based on the level and type of medical care that is provided, with inpatient services offering medical refeeding and monitoring which is not commonly offered at residential care centres (Twohig, Bluett, Torgesen, Lensegrav‐Benson, & Quakenbush‐Roberts, 2015). Residential care is generally offered to individuals who are medically stable but in need of a higher level of treatment intensity than that offered in outpatient settings (Twohig et al., 2015).

Residential treatment centres provide full‐time housing and multi‐disciplinary treatment in a non‐hospital‐based treatment setting. Treatment normally includes individual and group therapy components, meal support and various forms of recreational activities (Friedman et al., 2016). For individuals who are medically stable but require more intensive care than is offered by out‐patient services, residential care may provide a valuable bridge between hospital‐based inpatient treatment and traditional outpatient services (Thompson‐Brenner, Boswell, Espel‐Huynh, Brooks, & Lowe, 2018).

Most treatment research has focused on treatment outcomes at the individual inpatient or outpatient level, and there is a relative scarcity of studies investigating the effectiveness of eating disorder treatment at the residential level of care. The limited data in this area is concerning given the recent increase in residential care providers delivering services with yet to be determined efficacy (Guarda & Attia, 2018). In addition, although substantially less expensive than inpatient hospital care, residential care is more expensive to the community and patients than outpatient services (Frisch, Herzog, & Franko, 2006). Consequently, it is important to understand treatment outcomes in this setting. One valuable review of residential program was conducted by Friedman et al. (2016). They concluded that outcomes at discharge appeared favourable but noted study design limitations and the paucity of follow‐up data. However, since this review a further 12 studies have been reported which allows for a more extensive evaluation of outcome variables, predictors of treatment and follow‐up. Thus, the purpose of this report was to conduct a systematic review and qualitative synthesis of research that has evaluated outcomes of residential treatment for eating disorders across all diagnoses, age groups and genders. A secondary goal was to identify the treatment elements and patient characteristics that predicted a greater response to treatment.

## 2 METHODS

### 2.1 Search strategy

A systematic review was conducted in accordance with the PRISMA guidelines for systematic reviews (Moher, Liberati, Tetzlaff, & Altman, 2009). The databases PubMed and Cochrane Library were searched to identify peer‐reviewed studies examining the outcome of residential treatment programmes for adolescents and adults with eating disorders. Search terms included: ‘feeding and eating disorders’ or ‘eating disorder’ or ‘anorexia’ or ‘bulimia’ or ‘binge eating disorder’ or ‘OSFED’ or ‘EDNOS’ along with ‘residential’. Retrieval was limited to English language documents published over the 20‐year period between July 1999 and July 2019. Supplementary searches were conducted in Google Scholar using the ‘related articles’ field applied to key literature and by examining reference lists for articles not previously identified.

**Figure 1:**
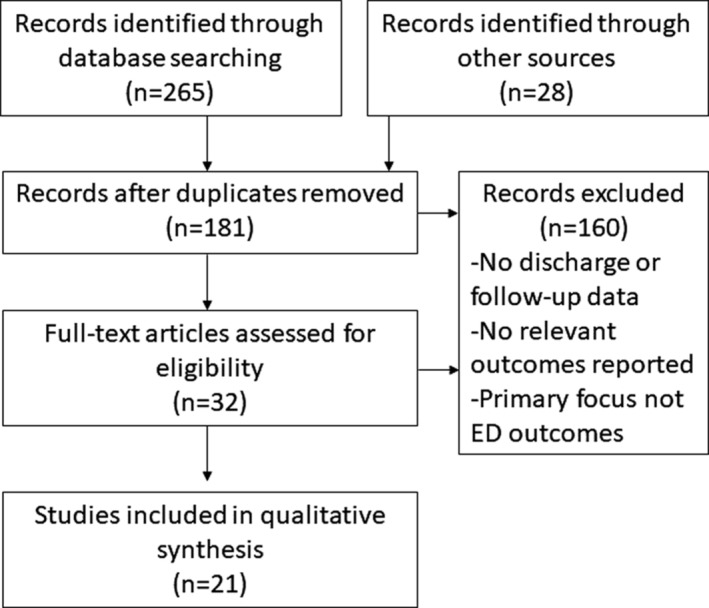
Flow chart of search strategy used to identify and screen relevant studies for review

Article titles and abstracts were screened initially, and then the full article content was reviewed for eligibility. Data were extracted when they met inclusion/exclusion criteria described below. Screening and review or entries was conducted by one author (T.P.). An overview of the literature search is displayed in Figure 1.

### 2.2 Inclusion/exclusion criteria

We included peer‐reviewed studies that reported on changes in at least one measure of eating disorder psychopathology or weight between intake and discharge and/or follow‐up as a primary outcome following residential treatment. Patients with any diagnosable eating disorder (according to the fourth or fifth edition of the Diagnostic and Statistical Manual of Mental Disorders) and any age or gender being treated in a residential setting were included in this review. Case reports (studies assessing less than three patients) and studies where treatment of eating disorders was not the primary focus, were excluded.

## 3 FINDINGS

### 3.1 Search results

We identified 19 studies that met the inclusion criteria for this review. All 19 studies were open‐label trials. None compared outcomes from a residential treatment with outcomes from a different treatment setting. One compared changes in eating disorder inventory (EDI; Garner, 2004) scores at the end of treatment to EDI scores in a non‐patient sample of college students, with clinical significance inferred if study patients were within the mean plus one standard deviation of the comparison group at discharge (Weltzin et al., 2007). Another study (Juarascio et al., 2013) compared outcomes in individuals who received acceptance and commitment therapy (ACT) in residential care with outcomes in individuals receiving residential care only (treatment as usual, TAU). Seventeen of the included studies were conducted at centres in the United States, and two were conducted in Italy.

### 3.2 Features of residential programs

Most of the studies in residential settings adopted an eclectic approach to treatment, integrating elements from several different techniques without a unifying theoretical framework. Amongst studies that provided details of their treatment program, residential treatment referred broadly to long‐term stays (typically >1 month) in 24‐hr care facilities designed specifically for patients with eating disorders, in which patients participated in multidisciplinary treatment programs integrating psychological therapy, nutritional management and medical management. Most programs included various forms of individual, group and family therapy, alongside a range of experiential activities, such as yoga, art or exercise. Treatment was delivered by a multidisciplinary team that included clinical psychologists, psychiatrists, master's level primary therapists, registered nurses, dieticians, family therapists and/or instructors for the various experiential activities (see Table 1).

**Table 1:** Percentage of programs reporting se of different types of therapy

Treatment modality	
CBT	52%
DBT	33%
ACT	19%
Exposure therapy	14%
Psychodynamic	10%
Feminist relational	10%
IPT	10%
Applied neuroscience	10%
Other treatment elements	
Group therapy	81%
Family therapy/programming	67%
Movement therapy	38%
Education support	33%
Ropes course	29%
Rewards program	24%
Meal prep	24%
Community reintegration	24%
Art	19%
Physical activity/exercise	19%
Yoga	19%
Recovered staff as role models	14%
Animal therapy	10%
Meditation	10%
Music	10%
Spirituality	10%

Abbreviations: ACT, acceptance and commitment therapy; CBT, cognitive behavioural therapy; DBT, dialectical behaviour therapy; IPT, interpersonal therapy.

Table 1 provides an overview of the treatment approaches and treatment elements described in the included literature, ranked by frequency. The most common treatment modalities were cognitive behavioural therapy (52% of studies), dialectical behavioural therapy (33%) and ACT (19%), followed by exposure therapy (14%), psychodynamic therapies, feminist relational therapy, interpersonal therapy and applied neuroscience (all 10%).

The length of stay varied widely across studies, ranging from 17 days (Lowe, Davis, Annunziato, & Lucks, 2003) to 377 days (Fortunato et al., 2017). The weighted mean length of stay across all studies that provided this information (*n* = 18 studies, 3,144 participants) was 56 days. Length of stay tended to be longer for individuals with AN compared with BN (weighted mean length of stay = 52 days for AN and 47 days for BN, *n* = 5 studies) and for males compared with females (weighted mean length of stay = 62 days for females and 67 days for males, *n* = 2 studies).

### 3.3 Participant characteristics

Inclusion criteria in residential treatment, when stated, were based on a diagnosis of AN, BN, BED or EDNOS by clinical interview (using DSM‐IV or DSM‐V criteria). Exclusion criteria were rare, but included patients who were medically unstable at admission (Bluett et al., 2016), patients with major psychiatric disorders, acute disease and/or a BMI <13 kg/m^2^ (Bonifacio et al., 2017), and previous admission during the data collection period of the study or residential stays <7 days (Thompson‐Brenner et al., 2018).

The majority of participants across all studies were Caucasian females. The weighted mean age was 24.4 years, with a range of 12–63 years. Participants with AN were included in all studies, whereas BN, BED and EDNOS/OSFED were represented in 90, 14 and 71% of studies respectively. Only a single study focused solely on male participants (Weltzin et al., 2012), who were otherwise represented in small numbers in 38% of studies.

Comorbidities were high in all studies that measured them, with 80–97% of participants observed to have at least one other psychiatric condition (Delinsky, 2010; Juarascio et al., 2013; Thompson‐Brenner et al., 2018; Weltzin, Bean, Klosterman, Lee, & Welk‐Richards, 2015; Weltzin et al., 2012). These included mood disorders (62–78% of individuals; Juarascio et al., 2013; Thompson‐Brenner et al., 2018; Weltzin et al., 2012), anxiety disorders (30–44%; Juarascio et al., 2013; Thompson‐Brenner et al., 2018; Weltzin et al., 2012), substance abuse or dependence (23–30%; Juarascio et al., 2013; Weltzin et al., 2012) and obsessive compulsive behaviours (20–22%; Weltzin et al., 2007).

Two studies noted that study participants had a high rate of prior hospitalisations. In one study, prior eating disorders‐related hospitalisations had occurred for 46–47% of individuals with AN and BN, respectively (Lowe et al., 2003). In a second study, 69% of participants reported prior psychiatric hospitalisations and 19% reported previous medical hospitalisations relating to an eating disorder or other psychiatric disorder (Delinsky, 2010).

In addition, one study observed that study participants were a treatment‐refractory group, with the majority of patients (90% of patients with AN and 93% of patients with BN) having failed to recover after previous outpatient, inpatient and/or residential treatment (Brewerton & Costin, 2011a). One study compared diagnostic groups at admission on eating disorder symptomatology scores [assessed with the Eating Disorder Examination Questionnaire (EDE‐Q)] and reported significantly higher scores in individuals with BN compared to those with AN (Fewell, Levinson, & Stark, 2017).

### 3.4 Summary of outcomes

Table 2 summarises the study characteristics and key findings of the included studies. All 19 studies reported a significant improvement between intake and discharge scores on at least one outcome measure. Significant improvement below refers to a statistically significant (*p* < .05) change, and the test used to determine a result is presented in brackets where relevant. Clinical significance was addressed in four studies, and variously defined as: scores within the mean plus one standard deviation of the community mean (Juarascio et al., 2013) or a non‐patient sample of college students (Weltzin et al., 2007); the midpoint between the study sample mean and non‐clinical sample mean (Thompson‐Brenner et al., 2018); and scores at post‐treatment that were equal to or greater than two standard deviations of improvement from the included sample mean (Twohig et al., 2016). Where the term clinical significance appears in the text below, we refer to the definition adopted by the original study authors.

**Table 2:** Summary of study characteristics and outcomes

Author, year, country	Sample at admission/EOT/follow‐up	Age (mean ± *SD* and range)	Gender(s); Diagnoses	Follow‐up	Length of stay in days (mean ± *SD*)	Therapeutic approach	Outcome (mean ± *SD*)			
							Measure	Intake	EOT	Follow‐up
Bean and Weltzin, 2001, USA	121/99/25	BN: 24 ± 8 (14–47) AN: 24 ± 11 (13–54)	Female; BN, AN	6 months	BN: 50 ± 25; AN: 72 ± 48	Multidisciplinary	EDI‐1 (total)	80.3 ± 50.2	42.3 ± 38.3***	Mean follow‐up scores for most subscales were similar to mean discharge scores
Bean et al., 2004, USA	33	Female: 22 ± 9 (12–45) Male: 18 ± 5 (13–29)	79% female; AN	15 months	Female: 72 ± 35; male: 84 ± 73	Multidisciplinary	BMI (female) BMI (male)	15 ± 2 16 ± 2	17 ± 2*** 19 ± 2*	18 ± 3* 21 ± 3*
Bluett et al., 2016, USA	113	19 ± 6 (12–45)	Female BN, AN, EDNOS			Multidisciplinary, including CBT, DBT, ACT and applied neuroscience	EDRC EDQOL BIAQ	52.5 ± 24.1 47.1 ± 19.8 36.9 ± 17.5	29.4 ± 22.6** 21.5 ± 19.7** 54.8 ± 16.5**	
Bonifacio et al., 2017, Italy	75/51/51	24 ± 4 (13–40)	Female; BN, AN, BED, EDNOS	1 month	BN: 150 ± 0 AN: 147 ± 21 BED: 255 ± 0 EDNOS: 147 ± 15	Multidisciplinary	BMI (AN) BMI (BN) BMI (BED) BMI (EDNOS)	15.2 ± 1.5 25.4 ± 6.8 45.3 ± 9.4 20.5 ± 4.9	18.1 ± 1.5* 24.7 ± 6 41.4 ± 8.6* 19.7 ± 1.3	18.6 ± 1.6* 24.8 ± 6.7 39.7 ± 8.4* 22.0 ± 4.8
Brewerton and Costin, 2011a, USA.	118	BN: 30 ± 7 (22–57) AN: 31 ± 9 (17–55)	Female; BN, AN	BN: 4 ± 3 years AN: 5 ± 3 years	BN: 83 ± 51 AN: 94 ± 52	Multidisciplinary (Monte Nido)	BMI (BN) BMI (AN) BDI (BN) BDI (AN)	20.6 ± 3.1 15.8 ± 1.6 27.4 ± 12.4 29.5 ± 12.2	21.2 ± 2.4 18.1 ± 1.4 9.6 ± 8.0† 13.5 ± 9.8†	20.8 ± 2.8 19.0 ± 3 .3***
							EDI‐2 (BN) EDI‐2 (AN)	11/11 subscales improve* 10/11 subscales improve*		
Brewerton and Costin, 2011b, USA	287/231	BN: 30 ± 8 (22–57) AN: 31 ± 8 (17–55)	Female; BN, AN		BN: 72 ± 45 AN: 98 ± 65		BMI (BN) BMI (AN)	20.8 ± 3.6 15.9 ± 1.7	21.6 ± 4.0 18.2 ± 1.4***	
							EDI‐2 (BN) EDI‐2 (AN)	11/11 subscales improve* 11/11 subscales improve*		
Delinsky, 2010, USA	80/36	19 ± 2 (13–23)	Female; BN, AN, EDNOS		BN: 44 ± 27 AN: 56 ± 26 EDNOS: 48 ± 27	Multidisciplinary, including CBT and DBT	EDE‐Q global BCQ BIAQ BDI QLESQ EBW (AN)	4.0 ± 1.3 71.6 ± 24.1 36.8 ± 9.9 30.5 ± 13.7 3.0 ± 0.7 81%	2.6 ± 1.4*** 59.9 ± 23.1*** 34.3 ± 10.2 16.4 ± 14.3*** 3.6 ± 0.7*** 92%†	
Espel, Goldstein, Manasse, Adrienne, & Hall, 2016, USA	53	30 ± 12 (18–63)	Female; BN, AN, EDNOS		29 ± 14	Multidisciplinary, including CBT, feminist‐relational group therapy and individual psychotherapy	EDE‐Q global	4.0 ± 1.6	2.3 ± 1.5***	
Fortunato et al., 2017, Italy	36/27	27 ± 6 (min age: 18)	97% female; AN		377 ± 143	Multidisciplinary	BMI MMPI (total) EDI‐3 (total) BUT‐GSI	15.2 620.5 439.3 2.6	17.4*** 581.5† 361.3† 2.0***	
Fewell et al., 2017, USA	423/–/65	24 ± 10 (11–60)	95% female; BN, AN, EDNOS, BED	12 months	50 ± 27 (includes PHP)	Multidisciplinary, including CBT and DBT	EDE‐Q CIA BMI BDI PSWQ WHO‐DAS	4.0 ± 1.5 33.9 ± 0.5 17.7 ± 0.2 31.4 ± 0.7 63.2 ± 0.6 2.3 ± 0.0	2.6 ± 1.5*** 20.6 ± 0.6*** 20.4 ± 0.2*** 19.3 ± 0.7*** 57.4 ± 0.7*** 1.9 ± 0.0***	2.9 ± 1.4*** 21.4 ± 0.6*** 20.6 ± 0.2***
Fitzpatrick and Weltzin, 2014, USA	107	24 ± 11	72% female; BN, AN, EDNOS		44 ± 23	Multidisciplinary	YBC‐EDS‐S YBC‐EDS‐M YBC‐EDS‐EGO	17.9 ± 7.4 9.4 ± 4.8 4.4 ± 2.4	16.6 ± 17.3*** 9.7 ± 5.0*** 4.1 ± 2.3***	
Juarascio et al., 2013, USA	105/–/65	29 ± 10 (18–55)	Female; BN, AN		27 ± 9	TAU: Includes psychodynamic, feminist, interpersonal, and CBT components TAU + ACT: Also includes twice‐weekly ACT group treatment	EDE‐Q global (all) EDE‐Q global (TAU) EDE‐Q global (TAU+ACT) BMI (BN) BMI (AN)	All subscales improve**		
								4.4 ± 1.2 4.3 ± 1.3 25.3 ± 7.7 17.2 ± 1.9	2.9 ± 1.3† 2.4 ± 1.3† 25.5 ± 8.3† 18.7 ± 2.1†	
Lee, Ong, Twohig, Lensegrav‐Benson, and Quakenbush‐Roberts, 2018, USA	142/103	19 ± 6 (12–45)	Female; BN, AN, OSFED		153 ± 65	Multidisciplinary, including CBT, ACT, DBT and applied neuroscience	BI‐AAQ AAQ‐II EDRC EDQOL OCI‐R BAI BDI‐II BMI	55.1 ± 17.4 30.0 ± 10.0 52.2 ± 24.0 46.5 ± 19.9 21.0 ± 15.4 23.2 ± 13.2 28.5 ± 14.0 19.5 ± 3.9	36.2 ± 16.0*** 20.2 ± 9.0*** 28.8 ± 22.3*** 21.1 ± 19.7*** 12.6 ± 13.2*** 11.5 ± 10.5*** 9.3 ± 11.7*** 21.9 ± 2.5***	
Lowe et al., 2003, USA	472/–/150	BN: 25 ± 8 AN: 24 ± 9 (13–61)	Female; BN, AN, EDNOS	6 months	AN: 19 BN: 17	Multidisciplinary, including psychodynamic, interpersonal and CBT therapies	*AN* BDI EAT‐D EAT‐O *BN* BDI EAT‐D EAT‐O	29.2 ± 10.5 23.6 ± 8.9 10.9 ± 4.7 29.1 ± 10.0 22.0 ± 8.5 4.8 ± 4.7	17.5 ± 10.6*** 15.1 ± 10.4*** 6.8 ± 4.7*** 18.3 ± 11.2*** 15.1 ± 9.2*** 2.6 ± 2.9***	20.2 ± 11.9*** 17.2 ± 10.9*** 6.8 ± 5.2*** 16.9 ± 12.9*** 14.4 ± 9.4** 3.3 ± 3.7**
Thompson‐Brenner et al., 2018, USA	440/–/266	25 ± 11 (13–63)	Female; AN, BN, BED, EDNOS/OSFED	6 months	TAU: 31 ± 15 UP: 27 ± 10	A transdiagnostic, common elements approach (the unified protocol for transdiagnostic treatment of emotional disorders or UP).	EDE‐Q (TAU) EDE‐Q (UP) CES‐D (TAU) CES‐D (UP) MEAQ (TAU) MEAQ (UP) SMQ (TAU) SMQ (UP) ASI (TAU) ASI (UP)	Sig refers to comparison between TAU and UP		
								4.1 ± 1.3 4.1 ± 1.4 37.1 ± 12.4 37.7 ± 12.1 229.2 ± 38.8 231.8 ± 40.4 31.0 ± 15.8 30.2 ± 15.9 32.3 ± 12.5 32.1 ± 12.8	2.5 ± 1.3 2.5 ± 1.2 25.8 ± 12.4 24.3 ± 11.7 212.2 ± 42.1 198.6 ± 46.4** 37.0 ± 15.9 42.6 ± 17.5** 30.6 ± 13.1 27.5 ± 13.3*	3.5 ± 1.6 2.5 ± 1.2*** 27.6 ± 134.4 17.7 ± 12.1*** 212.9 ± 43.9 161.1 ± 51.5*** 36.7 ± 16.4 34.8 ± 13.9 28.7 ± 12.1 25.6 ± 12.7
Twohig et al., 2016, USA	250/205	19 ± 6 (11–54)	Female; BN, AN, EDNOS			Multidisciplinary, including CBT, DBT and ACT	*Adolescents* BMI – AN BMI – BN BMI – EDNOS EDI‐3 (all dx)	16.8 ± 1.5 22.5 ± 2.4 20.0 ± 2.0	20.5 ± 1.2*** 23.1 ± 1.7*** 22.0 ± 1.7	
								3/3 subscales improve***		
							*Adults* BMI – AN BMI – BN BMI – EDNOS EDI‐3 (all dx)	16.8 ± 2.2 22.3 ± 4.5 22.9 ± 5.7	22.1 ± 1.5*** 23.1 ± 2.3** 24.0 ± 4.4	
								3/3 subscales improve***		
Weltzin et al., 2007, USA	384	Group 1: 22 + 8 Group 2: 23 + 9 Group 3: 23 + 8 Group 4: 20 ± 4	86% female; BN, AN, EDNOS		*Group 1*: ED only (F): 63 ± 39 *Group 2*: ED + OCB (F): 71 ± 34 *Group 3*: ED only (M):67 ± 28 *Group 4*: ED + OCB (M): 108 ± 54	Multidisciplinary, including psychotherapy	*EDI‐2 (total)* Group 1 Group 2 Group 3 Group 4	102 134 ± 39 73 97	59 ± 36* 85 ± 41* 40 ± 26* 51 ± 40*	
Weltzin et al., 2012, USA	111	Adults: 27 Adolescent: 15 (12–60)	Male; BN, AN, EDNOS		61 (8–166)	Multidisciplinary, including CBT	BMI (AN‐R) BMI (AN‐BP) BMI (BN) BMI (ENDOS) EDE‐Q global BDI STAI CAC	Mean change 3.5 ± 1.8*** 2.6 ± 2.0*** 0.8 ± 1.2** 0.0 ± 1.7 −1.6 ± 1.3** −13.6 ± 13.6** −20.8 ± 26** −4.8 ± 8.8**		
Weltzin et al., 2015, USA	145/–/50	Male: 23 ± 10 (16–58) Female: 23 ± 9 (13–53)	77% female; BN, AN, EDNOS	7 ± 4 months	M: 64 ± 32 F: 59 ± 25	Multidisciplinary	*EDQOL* Overall Males Females	103.8 ± 2.6 109.0 ± 26.1 98.8 ± 26.2	145.3 ± 2.7*** 155.3 ± 23.5 135.4 ± 29.1	147.8 ± 24 123.4 ± 35.9

Abbreviations; AAQ, Acceptance and Action Questionnaire; ACT, Acceptance and Commitment Therapy; AN, anorexia nervosa; ASI, Anxiety Sensitivity Index; BAI, Beck Anxiety Inventory; BCQ, Body Checking Questionnaire; BDI, Beck Depression Inventory; BED, binge eating disorder; BIAQ, Body Image Acceptance and Action Questionnaire; BMI, body mass index; BN, bulimia nervosa; BSQ, Body Shape Questionnaire; BUT‐GSI, Body Uneasiness Test‐Global Score Index; CAC, Compulsive Activity Checklist; CBT, cognitive behavioural therapy; CES‐D, Centre for Epidemiologic Studies‐Depression scale; CIA, Clinical Impairment Assessment; DBT, dialectical behaviour therapy; Dx, diagnosis; EAT, Eating Attitudes Test; EAT‐D, dieting subscale of EAT; EAT‐O, self‐control subscale of EAT; EBW, expected body weight; EDNOS, eating disorder not otherwise specified; EDRC, Eating Disorders Risk Composite; EDEQ, Eating Disorders Examination Questionnaire; EDI, eating disorders interview; EDQOL, Eating Disorders Quality of Life; EOT, end of treatment; MEAQ, The Multidimensional Experiential Avoidance Questionnaire; MMPI, Multiphasic Personality Inventory; OSFED, other specified feeding and eating disorder; PAI, Personality Assessment Inventory; PHP, partial hospitalisation program; PSWQ, Penn State Worry Questionnaire; QLESQ, Quality of Life Enjoyment and Satisfaction Questionnaire; SATAQ, Sociocultural Attitudes Towards Appearance Scale; *SD*, standard deviation; SMQ, Southampton Mindfulness Scale; STAI, State–Trait Anxiety Inventory; TAU, treatment as usual; WHO‐DAS, World Health Organization Disability Assessment Schedule; YBC‐EDS‐BSR, Yale‐Brown‐Cornell Eating Disorder Scale – Brief Self‐Report; YBC‐EDS‐M, motivation Subscale of YBC‐EDC; and YBC‐EDS‐EGO, egosyntonic Subscale of YBC‐EDS.

Significant values are denoted as follows: *p < .05, **p < .01, ***p < .001. †Refers to values in which the statistical significance has not been provided for a measure or time point.

#### 3.4.1 Eating disorders psychopathology

Sixteen studies reported changes in eating disorder psychopathology between intake and discharge. The EDI was the most commonly used assessment measure, administered in eight studies. Three studies used the EDE‐Q(Fairburn & Beglin, 2008), while all other measures were used in one to two studies each.

All 16 studies reported significant improvements in at least one outcome, including changes in ego‐syntonic behaviours, motivation to change, psychological flexibility, body image flexibility, obsessive–compulsive behaviours, psychological impairment scores, dieting, oral control, binge/purge behaviour, body image attitudes and compulsive behaviours.

Most studies observed similar levels of improvement across diagnostic groups and genders. However, one retrospective study reported that patients with AN had significantly better psychosocial functioning at discharge than patients with EDNOS (Fewell et al., 2017). Another study observed inferior outcomes in young adult females compared with males at discharge from a residential program (Weltzin et al., 2007).

Two studies compared standard residential care to residential care plus an additional program. A pilot study investigated the efficacy of a group‐based ACT treatment for 111 adult female residential patients with BN and AN compared with residential treatment only (TAU) (Juarascio et al., 2013). Large improvements were observed in almost all outcomes. A significantly larger portion of ACT+TAU patients had shifted from clinically significant ED symptoms at intake to the normative range at discharge compared with TAU patients (38 and 17%, respectively; *p* = .02). In addition, rehospitalizations following discharge were more likely to occur in the TAU group compared with the ACT + TAU group (18 and 3.5%, respectively).

Another study evaluated the preliminary effect of implementing a common elements therapy (Unified Protocol for Transdiagnostic Treatment of Emotional Disorders, or UP) on treatment outcomes in 440 adolescent and adult females ED patients attending residential care (Thompson‐Brenner et al., 2018). The UP model shares specific elements targeted for use across a range of co‐occurring emotional disorders with shared psychological features, and attempts to combine evidence‐based elements into a cohesive model. Comparisons were made between patients assigned to the pre‐implementation condition (TAU) and the UP model. At discharge, individuals in the UP group had higher mindfulness, lower anxiety sensitivity and lower experiential avoidance compared to TAU participants, although no significant differences were observed between groups in eating disorders or depressive symptoms. Both groups experienced significant improvements in experiential avoidance, anxiety sensitivity and mindfulness between intake and 6‐month follow‐up. Significantly more individuals in the UP group experienced clinically significant improvements in ED symptoms (EDE‐Q) between intake and follow‐up compared with the TAU group (65.5 and 34.9%, respectively).

#### 3.4.2 Body weight

Nine studies reported changes in weight between intake and discharge, with consistently positive outcomes. Amongst individuals with AN, BMI increased significantly from intake to discharge in all studies. One study reported that 39% of patients achieved weight recovery (BMI ≥18) by discharge which increased to 70% by follow‐up (Brewerton & Costin, 2011a). Another study reported that 80% of patients were discharged with a BMI of 18.5 or greater (up from 30% at admission). Similar results were reported in the other studies.

Individuals with BN had stable BMIs through the course of their treatment and discharge in three studies (Bonifacio et al., 2017; Brewerton & Costin, 2011a; Twohig et al., 2016) and increases in their BMI in three studies (Lee et al., 2018; Lowe et al., 2003; Weltzin et al., 2012).

Individuals with EDNOS maintained body weight through the course of treatment in one study (BMI of 20.5 ± 4.9 at entry and 22 ± 4.8 at follow‐up; Bonifacio et al., 2017) and experienced significant increases in BMI in another study (Twohig et al., 2016). A single study reported significant and progressive reductions in weight over the course of treatment and follow‐up in individuals with BED (Bonifacio et al., 2017).

#### 3.4.3 Depression, anxiety and quality of life

Nine studies reported improvements in depression scores amongst patients receiving residential care (Brewerton & Costin, 2011a, 2011b; Delinsky, 2010; Fewell et al., 2017; Fortunato et al., 2017; Lee et al., 2018; Lowe et al., 2003; Twohig et al., 2016; Weltzin et al., 2012). All nine studies included patients with AN, while a subset of studies additionally included patients with BN, BED and EDNOS/OSFED (8, 1 and 5 studies, respectively). One study reported significant improvements in depression that persisted through long‐term (mean 4.6 years) follow‐up (Brewerton & Costin, 2011a).

Five studies observed improvements in quality of life in individuals with BN, AN and EDNOS/OSFED (Bluett et al., 2016; Delinsky, 2010; Lee et al., 2018; Twohig et al., 2016; Weltzin et al., 2015).

Three studies reported improvements in anxiety amongst adolescent and adults with BN, AN and EDNOS/OSFED (Lee et al., 2018; Twohig et al., 2016; Weltzin et al., 2012). Another study reported a larger improvement in anxiety scores in males compared with females between intake and discharge as well as follow‐up (Weltzin et al., 2015).

#### 3.4.4 Follow‐up outcomes

Eight studies included follow‐up data collected between 1 month and 10 years after discharge. In seven of these studies the mean follow‐up period was 12 months or less; a single study included longer‐term follow‐ups with a mean of 4.6 years for patients with AN (Brewerton & Costin, 2011a).

Four studies reported that improvements in weight continued from discharge to follow‐up, with greater weights observed at follow‐up. One study reported an increase from 80% ideal body weight at discharge to 86% ideal body weight at 15‐month follow‐up, with greater weight gain observed in males (19 lbs) compared with females (7 lbs; Bean et al., 2004). A second study reported significant and progressive weight gain from treatment through one‐month follow‐up in individuals with AN, and progressive reductions in weight over the course of treatment and at follow‐up in individuals with BED (Bonifacio et al., 2017). A third study observed an increase in BMI from intake to discharge and follow‐up, with 70% achieving weight recovery (BMI ≥18) by follow‐up (Brewerton & Costin, 2011a). Similarly, a US study observed an increase in BMI between intake and discharge that was maintained at 1‐year follow‐up in 423 adolescent and adult patients with AN (Fewell et al., 2017).

Three studies reported improvements in eating disorders pathology at follow‐up. One study reported improvements in the drive for thinness, bulimia, ineffectiveness and interoceptive awareness subscales that were sustained from discharge to 6‐month follow‐up in 25 females with BN or AN (Bean & Weltzin, 2001). A second study reported significant improvements in eating disorders symptoms (EDI‐2) across 118 female participants with AN (10/11 subscales improved) and BN (all subscales) at long‐term follow‐up (mean: 4.6 years) (Brewerton & Costin, 2011a). A third study reported improvements between intake and 1‐year follow‐up in 423 adolescents and adults with BN, AN, BED and EDNOS, although ED symptomatology was significantly higher at follow‐up compared to discharge (Fewell et al., 2017).

Two studies observed improvements in depression at follow‐up. In one study, improvements occurred between intake and 3‐month follow‐up for adolescent and adult female participants with AN (Lowe et al., 2003), while the second study observed improvements lasting through long‐term follow‐up (mean: 4.6 years) in 188 females with BN and AN (Brewerton & Costin, 2011b).

Lastly, one study observed a significant increase in quality of life (EDQLS) between intake and discharge that persisted through follow‐up in males and females with BN, AN and EDNOS (Weltzin et al., 2015). A slight decrease in scores occurred at follow‐up, although outcomes remained significant.

#### 3.4.5 Subsequent treatment

Two studies reported on subsequent treatment for patients following discharge. One study reported that 60% of patients stepped down from residential to partial hospital treatment. Patients with AN had longer length of stay in partial hospital treatment than patients with BN and EDNOS (Delinsky, 2010). In another study, approximately 90% of individuals with AN and 80% of individuals with BN reported that they had continued in some form of outpatient treatment following discharge (Lowe et al., 2003). However, no detail is provided about the nature of outpatient treatment.

#### 3.4.6 Treatment adherence

Eight studies provided some information on the number of participants failing to complete treatment (Bean & Weltzin, 2001; Brewerton & Costin, 2011b; Delinsky, 2010; Fortunato et al., 2017; Lee et al., 2018; Lowe et al., 2003; Thompson‐Brenner et al., 2018; Twohig et al., 2016). Non‐completion was generally described as discharge outside of treatment plans and/or failure to complete discharge assessments, and ranged from 17 (Bean & Weltzin, 2001) to 66% (Lowe et al., 2003). One study detailed the reasons for drop‐out, and these included transferring to a different unit, such as for acute psychiatric stabilisation (9%); discharge against medical advice (8.5%); premature discharge initiated by medically stable individuals (2.5%); and premature discharge for violating program rules (2.5%; Delinsky, 2010).

Only two studies conducted intention‐to‐treat analyses, in both cases using the ‘last observation carried forward’ (LOCF) method (Juarascio et al., 2013; Lowe et al., 2003). Both studies reported that analyses conducted using LOCF were statistically equivalent to analyses conducted including non‐completers – that is, removing treatment non‐completers from the analyses did not alter outcomes.

Only two studies commented on the characteristics of non‐completers. In comparison to treatment completers with AN, one study found that non‐completers with AN were older, had a longer duration of illness, had less restricting behaviour and more frequent use of enemas (Brewerton & Costin, 2011b). A second study reported that non‐completers who were discharged against medical advice had shorter lengths of stay, lower % expected body weight (EBW) and more frequent previous hospitalisations (Delinsky, 2010).

Attrition between end of treatment (EOT) and follow‐up was high, ranging from 40 to 85% loss of participants by follow‐up (Bean & Weltzin, 2001; Bonifacio et al., 2017; Fewell et al., 2017; Juarascio et al., 2013; Lowe et al., 2003; Thompson‐Brenner et al., 2018; Weltzin et al., 2015).

### 3.5 Predictors of positive treatment outcomes

Several factors were found to predict residential treatment outcomes. These included the following variables.

- *Eating disorders pathology*. One study found that higher baseline eating disorders pathology was related to a failure to attain ≥90% EBW at discharge in females aged 16–23 with AN (Delinsky, 2010). A second study found that greater decreases in ED pathology predicted greater improvements in quality of life in males and females with BN, AN and EDNOS (Weltzin et al., 2015).
- *Depression and worry*. Three studies identified higher depression scores at intake as predictive of greater eating pathology at discharge. One study found that depression in females aged 16–23 with AN predicted ED pathology at discharge (Delinsky, 2010), while the second study found that depression and worry predicted ED symptomatology and psychological impairment at discharge and 1‐year follow‐up in adolescent and adult males and females with BN, AN, BED and EDNOS (Fewell et al., 2017). A third study similarly found that intake depression and worry predicted ED symptomatology and psychological impairment at discharge for individuals with AN, while worry predicted psychological impairment at 1‐year follow‐up individuals across all diagnosis (Fewell et al., 2017).
- *Obsessive–compulsive behaviours*. One study observed that eating disorders symptoms in young adult males and females with BN, AN and EDNOS at discharge were greater in individuals with comorbid OCB (Weltzin et al., 2007).
- *Body weight*. One study reported that lower EBW at intake was related to which participants would be discharged from residential care prematurely (against medical advice; Delinsky, 2010). A second study reported that admission BMI was significantly correlated with discharge BMI, with discharge BMI found to be the best predictor of full recovery from AN in this study (Brewerton & Costin, 2011a).
- *Experiential acceptance*. One study reported that higher levels of experiential acceptance (a willingness to tolerate or embrace aversive emotional experiences) at baseline predicted lower severity of ED symptoms at discharge (Espel, Goldstein, Manasse, Adrienne, & Hall, 2016). Individuals with higher levels of baseline acceptance tended to exhibit greater motivation for recovery, and subsequently experienced greater reductions in ED symptoms.
- *Motivation to change*. One study found that motivation to change significantly predicted eating pathology at discharge in 107 males and females with BN, AN and EDNOS (Fitzpatrick & Weltzin, 2014).
- *Body image flexibility*. One study found that an increase in body image flexibility over time was uniquely and significantly associated with lowered eating disorder risk, higher quality of life and improved mental well‐being, after accounting for changes in BMI, anxiety, depression and general psychological flexibility in adolescent and adult females with BN, AN and OSFED (Lee et al., 2018). Another study observed that after controlling for pre‐treatment symptoms and age, the level of eating disorder symptoms at the end of treatment (i.e., change in eating disorder symptoms) was predicted by greater psychological flexibility at intake (Bluett et al., 2016).
- *Psychosocial functioning*. One study found that psychosocial functioning at intake predicted ED symptomatology and psychological impairment in individuals with AN at 1‐year follow‐up (Fewell et al., 2017).
- *Length of stay*. One study observed that longer stays were associated with a greater reduction in ED and depressive symptoms (Thompson‐Brenner et al., 2018).

## 4 DISCUSSION

The primary purpose of this review was to evaluate behavioural and weight outcomes following residential treatment for eating disorders. To that end, we consistently found significant improvements in a range of outcomes across all individuals, including ED psychopathology, disordered eating behaviours, depression, anxiety, quality of life and body weight. These findings suggest that residential treatment can be effective at many levels, across diagnoses, genders and age groups.

However, given that symptom remission is a common prerequisite for discharge (Friedman et al., 2016), some level of improvement in symptoms can be expected. Improvements in weight, in particular, can be anticipated since food consumption is closely monitored and regulated in residential facilities. More meaningful data may come from measures of ‘internal’ cognitive processes, such as changes in psychopathology (Twohig et al., 2016). In support of residential setting treatments, improvements in psychopathology were observed across a wide range of measures. These included improvements in body image flexibility, psychological flexibility, obsessive–compulsive behaviours, weight concerns, motivation to change, depression, quality of life and all subscales for eating disorders psychopathology.

While these outcomes are ostensibly positive, there are numerous reasons why these data should be interpreted with caution. The most concerning is the lack of control groups comparing residential care to treatment at other levels, modalities or to no‐treatment controls. Randomised controlled trials (RCTs) are broadly recognised as the gold standard for evaluating the effectiveness of interventions. Recognising that RCTs are very hard to conduct in studies of eating disorder treatment, the next best option would be an equivalent control group receiving treatment in a different setting or no treatment. No studies of this kind are available and the lack of control groups makes it impossible to know how much of the improvement observed was due to the residential treatment itself. Although the severity and unremitting nature of eating disorders in residential participants makes it unlikely that the observed improvements were simply due to chance, there is little more that we can conclude without further research using more systematic study designs.

A further reason for caution is the extremely high drop‐out rate. The loss of participants between intake and discharge and discharge and follow‐up was substantial, ranging from 17–66% to 40–85%, respectively. Further, there has been an insufficient examination of the characteristics of treatment dropouts. Although high dropout rates are a problem in treatment research in the field generally, it substantially reduces confidence in the findings.

Eight studies included follow‐up data collected between 1 month to 10 years after discharge. In most cases, improvements observed at discharge tended to be sustained at follow‐up. Interestingly, while improvements in eating disorders pathology, depression and quality of life tended to persist (or slightly decrease) from discharge to follow‐up, weight tended to continue to improve between discharge and follow‐up. These outcomes suggest that for the subset of patients who complete treatment and follow‐up, residential treatment may lead to lasting physical and psychological improvements, which is undoubtedly positive. However, as with the baseline to discharge analyses, there are crucial design concerns. In particular, as mentioned above, there is a very substantial dropout at discharge and follow‐up. Although all studies included comparisons between intake and discharge, only two studies performed intention‐to‐treat analyses of treatment completers and non‐completers (Brewerton & Costin, 2011b; Delinsky, 2010). Without this vital comparison, it is impossible to know if treatment completers differ from non‐completers, and whether the omission of non‐completers from analyses have impacted the resulting outcomes.

Several baseline factors were identified that predicted treatment outcomes. Higher levels of eating disorders psychopathology, depression and worry at baseline were associated with inferior outcomes at discharge; lower body weight and intake was related to lower body weight at discharge and predicted which patients would be discharged prematurely; while higher levels of body image flexibility, psychosocial functioning, experiential acceptance and willingness to change were all associated with more positive treatment outcomes. Although each factor was discussed in only 1–2 studies, a recent systematic review evaluating predictors across a range of treatment settings and modalities identified many of the same predictors noted here (Vall & Wade, 2015).

### 4.1 Limitations of reviewed research

Residential care for eating disorders is increasingly common, yet there is a relative scarcity of evidence evaluating treatment outcomes. We identified only 19 studies providing intake to discharge outcome data over a 20‐year period – a drop in the bucket in comparison to the extensive evidence on outpatient interventions. As indicated above, the lack of effective control conditions represents a major limitation to the interpretation of studies such that it is not possible to recommend this treatment setting above another at this stage.

Another limitation is that the participant sample across most studies was relatively homogenous, comprised largely of Caucasian women. Additionally, 17 of the 19 studies were conducted in residential centres in America. These factors may limit the generalisability of this data to other populations.

Only a subset of the included studies conducted follow‐up assessments and these were often collected from a population that was substantially smaller than that at intake or discharge. Additionally, most follow‐up periods were short relative to the protracted nature of eating disorders, and may not have captured the full trajectory of symptom change. Research indicates that treatment outcomes at discharge may not be maintained at follow‐up (Carter et al., 2012; Friedman et al., 2016), and indeed may not even be predictive of recovery status longer‐term (Lock et al., 2013). Thus, relying on discharge data alone to determine the efficacy of treatment is problematic, and this is particularly the case for treatment programs that require a predetermined level of improvement prior to discharge (Friedman et al., 2016).

Another concern is that none of the studies included a detailed examination of the course of patients' treatment and recovery journey following residential care. Patients may have undergone additional treatments (or had life experiences that could have altered the course of their recovery) in the period between discharge from residential care and follow‐up, which may have impacted outcomes measured at follow‐up. It is therefore unclear the extent to which follow‐up outcomes should be attributed to residential care or other interim treatments.

The residential programs evaluated in this review varied widely in treatment models, assessment protocols, patient characteristics, follow‐up periods and average lengths of stay, which limits the generalizability of these findings. Many of these studies adopted an eclectic approach to treatment, combining various techniques, components and theoretical frameworks. Yet few studies provided detailed information regarding the theoretical framework used, the rationale for using it, and the treatment components used across different patient subpopulations. As such, it is impossible to draw conclusions on which treatment modalities or components were most effective in eliciting positive outcomes. Future studies that evaluate which aspects of a multidisciplinary program are the most meaningful, rather than validating a treatment package as a whole, would be beneficial in building evidence‐based residential treatment programs.

From a methodological perspective, one limitation of this study was that title and abstract screening and initial review of extracted papers were only conducted by one author. However, supplementary searches and examination of reference lists are likely to have largely overcome this limitation.

### 4.2 Conclusions

While the findings in this review suggest improvements following residential treatment, the poor quality of research designs prevents firm conclusions from being drawn. While the barriers implicit to RCTs are difficult to overcome, it may be more feasible for future research to prioritize retaining participants – a priority area given the low rates of retention through follow‐up. Future research should also include controlled studies that evaluate specific theoretical approaches and program elements in the residential setting, include long‐term follow‐up, and compare residential care to other treatment settings.

## CONFLICT OF INTEREST

The authors declare that there are no potential conflicts of interest with respect to the research, authorship, and/or publication of this article. Dr. Peckmezian received funding from the Butterfly Foundation to prepare this review, however, the authors alone were responsible for the content and writing of this paper.
